# Revisiting the personal protective equipment components of transmission-based precautions for the prevention of COVID-19 and other respiratory virus infections in healthcare

**DOI:** 10.2807/1560-7917.ES.2023.28.32.2200718

**Published:** 2023-08-10

**Authors:** Diamantis Plachouras, Oliver Kacelnik, Jesús Rodríguez-Baño, Gabriel Birgand, Michael A Borg, Brian Kristensen, Jan Kubele, Outi Lyytikäinen, Elisabeth Presterl, Jacqui Reilly, Andreas Voss, Walter Zingg, Carl Suetens, Dominique L Monnet

**Affiliations:** 1 European Centre for Disease Prevention and Control, Stockholm, Sweden; 2 Norwegian Institute of Public Health, Oslo, Norway; 3 Infectious Diseases and Microbiology division, Hospital Universitario Virgen Macarena and Department of Medicine, University of Seville/CSIC, Biomedicine Institute of Seville, Seville, Spain; 4 CIBERINFEC, Instituto de Salud Carlos III, Madrid, Spain; 5 Regional Centre for Infection Prevention and Control, Region of Pays de la Loire, Nantes, France; 6 Health Protection Research Unit, Healthcare Associated Infection and Antimicrobial Resistance, Imperial College London, London, United Kingdom; 7 Infection Control Department, Mater Dei Hospital, Msida, Malta; 8 Statens Serum Institut, Copenhagen, Denmark; 9 Clinical Microbiology and ATB centre, Na Homolce Hospital, Prague, Czechia; 10 Finnish Institute for Health and Welfare, Helsinki, Finland; 11 Department for Hospital Epidemiology and Infection Control, Medical University of Vienna, Vienna, Austria; 12 Research Centre for Health, Glasgow Caledonian University, Glasgow, United Kingdom; 13 Department of Medical Microbiology and Infection Prevention, University Medical Centre Groningen, Groningen, the Netherlands; 14 Department of Infectious Diseases and Hospital Epidemiology, University Hospital Zurich, Zurich, Switzerland; 15 Charité Universitätsmedizin, Berlin, Germany

**Keywords:** COVID-19, transmission-based precautions, infection prevention and control

## Abstract

The COVID-19 pandemic highlighted some potential limitations of transmission-based precautions. The distinction between transmission through large droplets vs aerosols, which have been fundamental concepts guiding infection control measures, has been questioned, leading to considerable variation in expert recommendations on transmission-based precautions for COVID-19. Furthermore, the application of elements of contact precautions, such as the use of gloves and gowns, is based on low-quality and inconclusive evidence and may have unintended consequences, such as increased incidence of healthcare-associated infections and spread of multidrug-resistant organisms. These observations indicate a need for high-quality studies to address the knowledge gaps and a need to revisit the theoretical background regarding various modes of transmission and the definitions of terms related to transmission. Further, we should examine the implications these definitions have on the following components of transmission-based precautions: (i) respiratory protection, (ii) use of gloves and gowns for the prevention of respiratory virus infections, (iii) aerosol-generating procedures and (iv) universal masking in healthcare settings as a control measure especially during seasonal epidemics. Such a review would ensure that transmission-based precautions are consistent and rationally based on available evidence, which would facilitate decision-making, guidance development and training, as well as their application in practice.

## Background

Standard and transmission-based precautions are essential elements of infection prevention and control (IPC) practices [1,2]. Transmission-based precautions are applied in addition to standard precautions in patients suspected or confirmed to be infected or colonised with certain microorganisms. There are three types of transmission-based precautions, depending on the transmission route: contact, droplet and airborne precautions. Each type of precautions has implications for the use of personal protective equipment (PPE) and for administrative and engineering control measures such as the placement of the patient, staff cohorting and training and environmental cleaning. Contact precautions include the application of gloves and gowns, droplet precautions require the use of medical face masks, and airborne precautions require the placement of the patient in an airborne precaution isolation room and the use of a well-fitted respirator by the staff [1]. Sometimes, transmission-based precautions are combined to ensure that multiple modes of transmission are addressed. For respiratory infections, two concepts of transmission through the air have been distinguished: short-range droplet transmission and long-range aerosol transmission. In the current transmission-based precautions, most respiratory viruses were considered as being transmitted primarily through large droplets and only in exceptional situations by aerosols, resulting in the widespread application of droplet precautions such as the use of a medical face masks, rather than precautions against airborne transmission [3,4]. The COVID-19 pandemic highlighted a potential limitation of the underpinning theory and application of transmission-based precautions as used during the past decades.

## Evidence for transmission routes and respective precautions

Emerging scientific evidence and revisiting previously available studies support the argument that the distinction between droplets and aerosols is artificial, and dogmatic size thresholds for ‘large droplets’ and ‘aerosols’ are not fully consistent with the physical properties that are relevant for the transmission of respiratory viruses [5-7]. While some evidence of the contribution of aerosols to the transmission of respiratory viruses, such as influenza, has been available, airborne transmission of influenza is still debatable and has not been considered a primary route [8-10]. Accumulating evidence from the COVID-19 pandemic indicated that aerosols may play a role in the transmission of severe acute respiratory syndrome coronavirus 2 (SARS-CoV-2) [5,11], thus leading to a review of the recommendations such as ventilation and questioning the rationale for droplet precautions to prevent transmission of SARS-CoV-2.

Only a few microorganisms, such as *Mycobacterium tuberculosis,* and the measles and varicella-zoster viruses, have traditionally been considered as being transmitted obligatorily or preferentially through the airborne route, thus necessitating precautions against airborne transmission, including the use of respirators and hospitalisation in single rooms with negative pressure [2]. These recommendations are based on limited evidence of long-distance transmission to people not in close proximity to the case. Based on observational studies of severe acute respiratory syndrome (SARS), respiratory viruses have also been considered to be transmitted opportunistically through aerosols during procedures that are associated with increased production of infectious aerosols, although the evidence is weak and often contradictory [3,12].

Altogether, the relevant role of various routes of transmission, which includes the physical transport of an infectious particle leading to infection, has been challenging to assess. Efforts to define criteria of proof for each transmission route have been useful but it is difficult to draw any conclusion with certainty [13,14]. The available evidence originates mostly from outbreak investigations and is rarely definitive proof of one route over another, as alternate explanations for transmission cannot be dismissed nor can the adherence to the protective measures be easily assessed. The persistence of viruses on environmental surfaces and aerosols has also been extensively tested [15]. Few human exposure studies have been performed to elucidate the route of transmission of some respiratory viruses, and the results have often been contradictory, and it is uncertain if they can be extrapolated to other viruses [14,16]. These results indicate that several routes contribute to the transmission of respiratory viruses, depending on various factors such as the viral loads, presence of virus in secretions, activities and behaviour of the infected and exposed persons. For example, singing is an activity that was associated with a well-documented SARS-CoV-2 outbreak in a choir, presumably through increased release of respiratory secretions [17]. In addition, differences in expelled viral loads have also been hypothesised as explanations of superspreading events of SARS-CoV-2, where transmission was linked to a few highly infectious individuals [18]. 

Furthermore, the quality and certainty of evidence on the effectiveness of various elements of PPE used for transmission-based precautions remains low, as the evidence is based mostly on observational studies and only a few randomised controlled trials. Several systematic reviews and meta-analyses have been published but the certainty of the conclusions is limited by the bias of the underlying studies [19]. This means that guidance is, to a large extent, based on assumptions about the transmission routes, indirect evidence, feasibility, availability of resources and the precautionary principle. One example is the lack of convincing evidence in epidemiological studies or clinical trials that respirators should have a better protective effect against SARS-CoV-2 transmission than medical face masks, despite the better filtration efficacy and fitting of respirators [20-22]. Given these uncertainties, there has been considerable variation in expert recommendations on transmission-based precautions for COVID-19 in healthcare settings. One example of this is that some countries have opted for medical face masks, whereas other countries recommended respirators for the routine care of suspected and/or confirmed COVID-19 patients [23]. Because of the lack of evidence, individual values and preferences may play an important role in selecting the type of face mask, as also acknowledged in the guidance from the World Health Organization [24]. Furthermore, while transmission of SARS-CoV-2 through direct and indirect contact has not been demonstrated convincingly, replication-competent SARS-CoV-2 has been detected on environmental surfaces and objects [15]. Therefore, fomite transmission is considered possible and remains an important consideration when deciding on the need for contact precautions, such as the use of gowns and gloves.

Later variants of SARS-CoV-2 are characterised by higher transmissibility than previous variants [25]. On the other hand, severity of disease has been reported to be lower, possibly related to intrinsic viral characteristics as well as higher population immunity due to vaccination and previous infection, and thereby better protection against severe disease. Nevertheless, patients in healthcare facilities are often vulnerable due to their age, comorbidities and immunosuppressive treatment. Transmission from COVID-19 cases (patients, staff members or visitors) is an ongoing risk, especially when the prevalence of COVID-19 in the community is high. During the pandemic, universal masking in healthcare settings (medical face mask for all persons – staff, patients, visitors, service providers and others – within the health facility, including primary, secondary and tertiary care levels, outpatient care and long-term care facilities) has been applied extensively to minimise this risk, although the evidence base for this measure remains weak [26]. Well-designed studies of the effectiveness of universal masking during winter epidemics of respiratory viral illnesses are required.

These observations indicate a need to revisit transmission-based precautions and their effectiveness for preventing transmission of SARS-CoV-2 in particular and other respiratory viruses in general, as well as a need to fund, design and execute high-quality studies to fill the evidence gaps for transmission-based precautions [27].

## Which precautions do we need to revisit? Opinion of the ECDC expert panel

On 16 March 2022, the European Centre for Disease Prevention and Control (ECDC) convened a group of 11 IPC experts to explore whether modifications in transmission-based precautions would be needed to address what we learned during the COVID-19 pandemic and to identify knowledge gaps and research needs. Experts were selected as a convenience sample from the ECDC Expert Directory based on declared expertise in hygiene and infection control. The discussion was structured based on the following elements of the Evidence to Decision (EtD) framework [28]: (i) How substantial are the desirable anticipated effects? (ii) How substantial are the undesirable anticipated effects? (iii) What is the overall certainty of the evidence of effects? (iv) Does the balance between desirable and undesirable effects favour the option or the alternative? (v) Is the option acceptable to key stakeholders? (vi) Can the option be easily implemented? The following topics were discussed on the basis of lack of current consensus: (i) respiratory protection, (ii) use of gloves and gowns, (iii) aerosol-generating procedures and (iv) universal masking. Through online polling, the experts in the panel rated the EtD elements related to each of these topics. The results of the poll were used to guide the discussion. The group proposed that the following elements of the current transmission-based precautions be reviewed, ideally with support from well-designed applied clinical research (Box).

Box **Proposed points for review and research gaps related to the transmission-based precautions framework for respiratory viral infections**

- A review of the theoretical framework, and definitions of terms, for modes of transmission underpinning the IPC paradigm;
- Risk assessment parameters for IPC measures based on risk of infection transmission inclusive of: transmissibility of infection, impact of infection, patient factors, staff risk factors, practice/procedure factors and environmental factors, while taking into consideration: healthcare workers’ preference, desirable and undesirable anticipated effects and the balance between them, overall certainty of the evidence of effects, acceptability and ease of implementation;
- Design and implementation of high-quality applied clinical research to inform: - The use of respirators and medical face masks in various risk scenarios, - The impact of gowns, aprons and eye protection as part of transmission-based precautions, - Optimal masking strategies to reduce risk of transmission inclusive of sessional vs risk-targeted, wider masking policy, including behavioural insights to adherence, any unintended consequences for staff and patient safety, and staff preferences, - A risk-based approach to the selection of personal protective equipment considering the time of exposure, the proximity to the patient and the procedure/task.

IPC: infection prevention and control.

### Respiratory protection

Given that neither the current droplet nor the airborne precautions address the role of aerosols at short distances, various options could be considered for adapting current transmission-based precautions. Droplet precaution measures could be expanded to address the theoretical risks linked to inhalation of short-range aerosols. A two-tiered risk-based approach could be applied, based on proximity, length of exposure, type of symptoms and type of care: (i) contacts with prolonged, close proximity to the patient, including the performance of high-risk procedures, are associated with higher risk and justify the use of high-efficacy respiratory protection; (ii) for contacts without prolonged, close proximity to an infectious patient in adequately ventilated spaces, the effectiveness of medical face masks would be sufficient.

### Gloves and gowns

Gloves are part of contact precautions that have been recommended for the prevention of the transmission of respiratory viruses such as influenza and SARS-CoV-2. However, hand hygiene, including alcohol-based hand rubs, is an effective measure against SARS-CoV-2 and other respiratory viruses [29,30], as well as other microorganisms causing healthcare-associated infections [31]. It is a key component of standard IPC precautions. Wearing gloves has not been demonstrated to be superior to hand hygiene and has been associated with poor hand hygiene practices [32,33]. Gowns or aprons are also recommended as a measure to prevent contamination of clothes with the potential risk of subsequent transmission. However, there is limited and low-quality evidence to support the effectiveness of gloves, gowns, and aprons for the prevention of respiratory tract infections or the superiority of gowns vs aprons [3,21,30,32,34-36]. Dedicated footwear or shoe covers are not recommended for routine care [37].

While the desirable anticipated effects from the use of gloves and gowns/aprons are small, the undesirable anticipated effects are at least moderate. Therefore, the balance of desirable and undesirable effects does not favour the routine use of gloves or gowns/aprons for the prevention of COVID-19 and other respiratory virus infections. An exception may be prolonged, close exposure to a case, especially a patient who is coughing or sneezing heavily, when high-risk procedures are required, or in cases when there is a high risk of exposure to infectious body fluids - as stipulated by standard precautions.

### Aerosol-generating procedures

There is emerging evidence that the increased risk of infection from some aerosol-generating procedures may be more related to proximity with the patient than to increased generation of aerosols by the procedure itself [38]. Several studies have failed to demonstrate substantial aerosol generation linked to procedures such as intubation and extubation, for which the risk for transmission of respiratory viruses such as SARS had been shown to be high [39,40]. This suggests that the term ‘aerosol-generating procedures’ may be a misnomer and could be replaced by alternative terminologies such as ‘high-risk procedures’, to reflect this emerging evidence base. Further clinical research is needed to better describe the risks associated with these procedures and the role of aerosol generation in infection transmission.

Given that the risk of transmission is related not only to the microorganism, but also to the duration and proximity of contact, the applied transmission-based precautions should ideally be selected following a point-of-care risk assessment, taking into account proximity, duration of exposure, risk of aerosol production and the type of task performed.

### Universal masking

Various forms of continuous face mask use, such as universal masking, applying to healthcare workers, patients, visitors and persons accompanying the patient, and targeted continuous masking, applying to healthcare workers [26], especially during periods of high community transmission of respiratory viruses, such as the influenza season and during COVID-19 surges, are a potential measure to minimise healthcare-associated transmission of respiratory viruses [41]. The rationale for wearing a face mask includes both personal protection and source control, i.e. limiting the release of infectious respiratory particles by people who are infected and often asymptomatic or with limited symptoms. Given the risk of transmission of other respiratory viruses, such as influenza, and the role of other respiratory viruses in healthcare-associated pneumonia [42], it is likely that universal masking and targeted continuous masking could contribute towards preventing the transmission of other respiratory viruses in healthcare settings during seasonal epidemics.

### Eye protection

The eyes have been identified as potential routes of entry for respiratory viruses either directly, by infection of the ocular mucosa, or by transfer to the nasopharynx through the nasolacrimal duct [43]. Available evidence shows that eye protection is effective in decreasing the risk of transmission [44].

## Discussion

A limitation of the above suggestions is the low level of available evidence in epidemiological studies or clinical trials for the effectiveness of specific elements of transmission-based precautions, including elements of PPE. Emerging evidence may increase our certainty in the magnitude of their effectiveness. A better understanding of the physical processes of exposure does not automatically translate into more effective prevention measures, as infection is a more complicated process than the transfer of infectious particles, involving immune processes and the suitability of various exposed host tissues for the entry of viruses. The applied preventive measures aim at mitigating the risk of infection to an acceptable level, taking into account the assessed risk but also other parameters such as preferences, ease of implementation and unintended consequences. Studies which use various forms of modelling and laboratory experiments to investigate the role of procedures and behaviours in generating and dispersing respiratory particles are limited and ultimately do not provide adequate evidence to address transmission in the complex healthcare environment. There is a need for applied clinical research on the effectiveness but also the unintended consequences of preventive measures to support the development of evidence-based recommendations for guidance. Furthermore, human challenge studies, taking carefully into consideration any ethical issues [45], may provide valuable insights in the relative contribution of various routes of transmission and the effectiveness of PPE [46].

Transmission-based precautions are important but are not the only measures to control SARS-CoV-2 transmission in healthcare. It should be noted that PPE is only one among several control measures in the hierarchy that includes elimination and substitution, engineering and administrative controls [47]. Transmission of SARS-CoV-2 can occur before the primary case is suspected or diagnosed and a person can be more infectious at the early stages of disease. Once the clinical condition deteriorates – usually after the first week of disease – and the patient presents to hospital, the amount of shed SARS-CoV-2 is usually reduced, and the patient is consequently less infectious [48]. However, the duration of infectiousness can be affected by factors such as an immunocompromised state [49], severity of disease [50] and previous vaccination or infection [51]. The highest risk for the transmission of SARS-CoV-2 in healthcare comes from unidentified cases during periods of high community prevalence, which can be addressed by high index of suspicion and timely testing to guide the application of preventive measures. Finally, healthcare workers more often get infected through unprotected interactions with colleagues, family members and other private contacts [36]. Several other factors may influence the effectiveness of PPE, including the fit of face masks and respirators [52] and breaches in their proper use. All these points indicate that control of SARS-CoV-2 transmission in healthcare facilities will remain a challenge even after further optimisation of transmission-based precautions.

Measures to prevent and control an infection with high morbidity and case fatality and without treatment options are influenced by the assessed risk of adverse outcome [53] and often include additional PPE such as head covers, shoe covers or coveralls despite limited evidence of effectiveness. Such additional PPE reduces wearer comfort and is linked to a greater likelihood of self-contamination during PPE removal [54]. The same applies to emerging infections with a high likelihood of transmission. The role of the assessed risk in the selection of IPC measures is not reflected in the current definitions of transmission-based precautions, which is based on a mechanistic approach to transmission routes. Any revision of the transmission-based precautions should ideally make this point explicit.

## Conclusion

The ECDC expert panel suggested that the IPC community should consider revisiting the current transmission-based precaution framework, including the theoretical background regarding various modes of transmission and the definitions of terms related to transmission, to account for available and emerging scientific evidence and the role that the consequence of infection plays on the choice of the precautions. Such a review would ensure that transmission-based precautions are consistent and rationally based on available evidence, which would facilitate decision-making, guidance development and training, as well as their application in practice to prevent transmission of SARS-CoV-2 and of other respiratory viruses. High-quality studies are imperative to address the current knowledge gaps and strengthen the evidence for any future revision of transmission-based precautions. 

## References

[1] World Health Organization (WHO). Prevention of hospital-acquired infections: a practical guide. 2^nd^ ed. Ducel G, Fabry J, Nicolle L, editors. Geneva: WHO; 2002. Available from: https://apps.who.int/iris/handle/10665/67350

[2] Siegel JD, Rhinehart E, Jackson M, Chiarello R, the Healthcare Infection Control Practices Advisory Committee. 2007 Guideline for isolation precautions: preventing transmission of infectious agents in healthcare settings. Atlanta: CDC; 2007. Available from: https://www.cdc.gov/infectioncontrol/ guidelines/isolation/index.html 10.1016/j.ajic.2007.10.007 PMC7119119 18068815

[3] World Health Organization (WHO). Infection prevention and control of epidemic- and pandemic-prone acute respiratory diseases in health care. Geneva: WHO; 2014. Available from: https://apps.who.int/iris/rest/bitstreams/515717/retrieve 24983124

[4] Roy CJ Milton DK . Airborne transmission of communicable infection--the elusive pathway. N Engl J Med. 2004;350(17):1710-2. . Available from: https://www.ncbi.nlm.nih.gov/pubmed/15102996 10.1056/NEJMp048051 15102996

[5] Tang JW . SARS-CoV-2 and aerosols-Arguing over the evidence. J Virol Methods. 2021;289:114033. 10.1016/j.jviromet.2020.114033 33285192 PMC7716743

[6] Randall K Ewing ET Marr LC Jimenez JL Bourouiba L . How did we get here: what are droplets and aerosols and how far do they go? A historical perspective on the transmission of respiratory infectious diseases. Interface Focus. 2021;11(6):20210049. 10.1098/rsfs.2021.0049 34956601 PMC8504878

[7] Association for Aerosol Research. Position paper of the Gesellschaft für Aerosolforschung on understanding the role of aerosol particles in SARS-CoV-2 infection. Köln: Gesellschaft für Aerosolforschung; 2020. Available from: https://www.info.gaef.de/_files/ugd/fab12b_0b691414cfb344fe96d4b44e6f44a5ab.pdf

[8] Tellier R . Review of aerosol transmission of influenza A virus. Emerg Infect Dis. 2006;12(11):1657-62. 10.3201/eid1211.060426 17283614 PMC3372341

[9] Tellier R . Aerosol transmission of influenza A virus: a review of new studies. J R Soc Interface. 2009;6(Suppl 6) Suppl 6;S783-90. 10.1098/rsif.2009.0302.focus 19773292 PMC2843947

[10] Brankston G Gitterman L Hirji Z Lemieux C Gardam M . Transmission of influenza A in human beings. Lancet Infect Dis. 2007;7(4):257-65. 10.1016/S1473-3099(07)70029-4 17376383

[11] van der Valk JPM In ’t Veen JCCM . SARS-Cov-2: the relevance and prevention of aerosol transmission. J Occup Environ Med. 2021;63(6):e395-401. 10.1097/JOM.0000000000002193 33871953 PMC8168703

[12] Leal J Farkas B Mastikhina L Flanagan J Skidmore B Salmon C Risk of transmission of respiratory viruses during aerosol-generating medical procedures (AGMPs) revisited in the COVID-19 pandemic: a systematic review. Antimicrob Resist Infect Control. 2022;11(1):102. 10.1186/s13756-022-01133-8 35953854 PMC9366810

[13] Jefferson T Heneghan CJ Spencer E Brassey J Plüddemann A Onakpoya I A hierarchical framework for assessing transmission causality of respiratory viruses. Viruses. 2022;14(8):1605. 10.3390/v14081605 35893670 PMC9332164

[14] Gwaltney JM Jr Hendley JO . Rhinovirus transmission: one if by air, two if by hand. Am J Epidemiol. 1978;107(5):357-61. 10.1093/oxfordjournals.aje.a112555 208415

[15] Onakpoya IJ Heneghan CJ Spencer EA Brassey J Rosca EC Maltoni S Viral cultures for assessing fomite transmission of SARS-CoV-2: a systematic review and meta-analysis. J Hosp Infect. 2022;130:63-94. 10.1016/j.jhin.2022.09.007 36115620 PMC9473144

[16] Dick EC Jennings LC Mink KA Wartgow CD Inhorn SL . Aerosol transmission of rhinovirus colds. J Infect Dis. 1987;156(3):442-8. 10.1093/infdis/156.3.442 3039011

[17] Hamner L Dubbel P Capron I Ross A Jordan A Lee J High SARS-CoV-2 Attack Rate Following Exposure at a Choir Practice - Skagit County, Washington, March 2020. MMWR Morb Mortal Wkly Rep. 2020;69(19):606-10. 10.15585/mmwr.mm6919e6 32407303

[18] Chen PZ Koopmans M Fisman DN Gu FX . Understanding why superspreading drives the COVID-19 pandemic but not the H1N1 pandemic. Lancet Infect Dis. 2021;21(9):1203-4. 10.1016/S1473-3099(21)00406-0 34352224 PMC8328407

[19] Murad MH Asi N Alsawas M Alahdab F . New evidence pyramid. Evid Based Med. 2016;21(4):125-7. 10.1136/ebmed-2016-110401 27339128 PMC4975798

[20] Chou R Dana T Jungbauer R Weeks C McDonagh MS . Masks for prevention of respiratory virus infections, including SARS-CoV-2, in health care and community settings: a living rapid review. Ann Intern Med. 2020;173(7):542-55. 10.7326/M20-3213 32579379 PMC7322812

[21] Jefferson T Del Mar CB Dooley L Ferroni E Al-Ansary LA Bawazeer GA Physical interventions to interrupt or reduce the spread of respiratory viruses. Cochrane Database Syst Rev. 2020;11(11):CD006207. 33215698 10.1002/14651858.CD006207.pub5 PMC8094623

[22] Loeb M Bartholomew A Hashmi M Tarhuni W Hassany M Youngster I Medical masks versus N95 respirators for preventing COVID-19 among health care workers: a randomized trial. Ann Intern Med. 2022;175(12):1629-38. 10.7326/M22-1966 36442064 PMC9707441

[23] Birgand G Mutters NT Otter J Eichel VM Lepelletier D Morgan DJ Variation of national and international guidelines on respiratory protection for health care professionals during the COVID-19 pandemic. JAMA Netw Open. 2021;4(8):e2119257. 10.1001/jamanetworkopen.2021.19257 34347062 PMC8339937

[24] World Health Organization (WHO). Infection prevention and control in the context of coronavirus disease (COVID-19): a living guideline. Geneva: WHO; 2023. Available from: https://apps.who.int/iris/bitstream/handle/10665/365576/WHO-2019-nCoV-ipc-guideline-2023.1-eng.pdf 35767666

[25] Lyngse FP Mortensen LH Denwood MJ Christiansen LE Møller CH Skov RL Household transmission of the SARS-CoV-2 Omicron variant in Denmark. Nat Commun. 2022;13(1):5573. 10.1038/s41467-022-33328-3 36151099 PMC9508106

[26] World Health Organization (WHO). Infection prevention and control during health care when coronavirus disease (COVID-19) is suspected or confirmed Geneva: WHO; 2021. Available from: https://apps.who.int/iris/bitstream/handle/10665/342620/WHO-2019-nCoV-IPC-2021.1-eng.pdf?sequence=1&isAllowed=y

[27] Glasziou PP Michie S Fretheim A . Public health measures for covid-19. BMJ. 2021;375(2729):n2729. 10.1136/bmj.n2729 34789507

[28] Moberg J Oxman AD Rosenbaum S Schünemann HJ Guyatt G Flottorp S The GRADE Evidence to Decision (EtD) framework for health system and public health decisions. Health Res Policy Syst. 2018;16(1):45. 10.1186/s12961-018-0320-2 29843743 PMC5975536

[29] Kratzel A Todt D V’kovski P Steiner S Gultom M Thao TTN Inactivation of severe acute respiratory syndrome coronavirus 2 by WHO-recommended hand rub formulations and alcohols. Emerg Infect Dis. 2020;26(7):1592-5. 10.3201/eid2607.200915 32284092 PMC7323537

[30] Jefferson T Dooley L Ferroni E Al-Ansary LA van Driel ML Bawazeer GA Physical interventions to interrupt or reduce the spread of respiratory viruses. Cochrane Database Syst Rev. 2023;1(1):CD006207. 36715243 10.1002/14651858.CD006207.pub6 PMC9885521

[31] World Health Organization (WHO). Guidelines on hand hygiene in health care. Geneva: WHO; 2009. Available from: https://apps.who.int/iris/rest/bitstreams/52455/retrieve

[32] Fuller C Savage J Besser S Hayward A Cookson B Cooper B "The dirty hand in the latex glove": a study of hand hygiene compliance when gloves are worn. Infect Control Hosp Epidemiol. 2011;32(12):1194-9. 10.1086/662619 22080658

[33] Cusini A Nydegger D Kaspar T Schweiger A Kuhn R Marschall J . Improved hand hygiene compliance after eliminating mandatory glove use from contact precautions-Is less more? Am J Infect Control. 2015;43(9):922-7. 10.1016/j.ajic.2015.05.019 26122873

[34] Loveday HP Wilson JA Pratt RJ Golsorkhi M Tingle A Bak A epic3: national evidence-based guidelines for preventing healthcare-associated infections in NHS hospitals in England. J Hosp Infect. 2014;86(Suppl 1):S1-70. 10.1016/S0195-6701(13)60012-2 24330862 PMC7114876

[35] Meda M Gentry V Reidy P Garner D . Unintended consequences of long-sleeved gowns in a critical care setting during the COVID-19 pandemic. J Hosp Infect. 2020;106(3):605-9. 10.1016/j.jhin.2020.07.036 32745589 PMC7832883

[36] Belan M Charmet T Schaeffer L Tubiana S Duval X Lucet JC SARS-CoV-2 exposures of healthcare workers from primary care, long-term care facilities and hospitals: a nationwide matched case-control study. Clin Microbiol Infect. 2022;28(11):1471-6. 10.1016/j.cmi.2022.05.038 35777605 PMC9239704

[37] Antimicrobial Resistance and Healthcare Associated Infection (ARHAI) Scotland Infection Control Team. Standard infection control precautions. Literature review. Personal protective equipment (PPE) footwear. Edinburgh: Public Health Scotland; 2021. Available from: https://www.nipcm.hps.scot.nhs.uk/media/1901/2021-07-22-ppe-footwear-v30-final.pdf

[38] Wilson NM Marks GB Eckhardt A Clarke AM Young FP Garden FL The effect of respiratory activity, non-invasive respiratory support and facemasks on aerosol generation and its relevance to COVID-19. Anaesthesia. 2021;76(11):1465-74. 10.1111/anae.15475 33784793 PMC8250912

[39] Tran K Cimon K Severn M Pessoa-Silva CL Conly J . Aerosol generating procedures and risk of transmission of acute respiratory infections to healthcare workers: a systematic review. PLoS One. 2012;7(4):e35797. 10.1371/journal.pone.0035797 22563403 PMC3338532

[40] National Health Service (NHS) England. A rapid review of aerosol generating procedures (AGPs). Leeds: NHS England. 2022. Available from: https://www.england.nhs.uk/wp-content/uploads/2022/04/C1632_rapid-review-of-aerosol-generating-procedures.pdf

[41] World Health Organization (WHO). Infection prevention and control in the context of coronavirus disease (COVID-19): a living guideline. Geneva: WHO; 2023. Available from: https://www.who.int/publications/i/item/WHO-2019-nCoV-ipc-guideline-2022.2 35767666

[42] Shorr AF Zilberberg MD Micek ST Kollef MH . Viruses are prevalent in non-ventilated hospital-acquired pneumonia. Respir Med. 2017;122:76-80. 10.1016/j.rmed.2016.11.023 27993295 PMC7135153

[43] Coroneo MT Collignon PJ . SARS-CoV-2: eye protection might be the missing key. Lancet Microbe. 2021;2(5):e173-4. 10.1016/S2666-5247(21)00040-9 33655228 PMC7906687

[44] Chu DK Akl EA Duda S Solo K Yaacoub S Schünemann HJ Physical distancing, face masks, and eye protection to prevent person-to-person transmission of SARS-CoV-2 and COVID-19: a systematic review and meta-analysis. Lancet. 2020;395(10242):1973-87. 10.1016/S0140-6736(20)31142-9 32497510 PMC7263814

[45] Jamrozik E Selgelid MJ . COVID-19 human challenge studies: ethical issues. Lancet Infect Dis. 2020;20(8):e198-203. 10.1016/S1473-3099(20)30438-2 32479747 PMC7259898

[46] Killingley B Mann AJ Kalinova M Boyers A Goonawardane N Zhou J Safety, tolerability and viral kinetics during SARS-CoV-2 human challenge in young adults. Nat Med. 2022;28(5):1031-41. 10.1038/s41591-022-01780-9 35361992

[47] Centers for Disease Control and Prevention (CDC). Hierarchy of controls. Atlanta: CDC. [Accessed: 28 Feb 2023]. Available from: https://www.cdc.gov/niosh/topics/hierarchy/default.html

[48] Puhach O Meyer B Eckerle I . SARS-CoV-2 viral load and shedding kinetics. Nat Rev Microbiol. 2023;21(3):147-61. 36460930 10.1038/s41579-022-00822-w PMC9716513

[49] Jefferson T Spencer EA Conly JM Rosca EC Maltoni S Brassey J Viral cultures, cycle threshold values and viral load estimation for assessing SARS-CoV-2 infectiousness in haematopoietic stem cell and solid organ transplant patients: a systematic review. J Hosp Infect. 2023;132:62-72. 10.1016/j.jhin.2022.11.018 36473552 PMC9721162

[50] Nomura T Kitagawa H Omori K Shigemoto N Kakimoto M Nazmul T Duration of infectious virus shedding in patients with severe coronavirus disease 2019 who required mechanical ventilation. J Infect Chemother. 2022;28(1):19-23. 10.1016/j.jiac.2021.09.006 34538728 PMC8429366

[51] Jung J Kim JY Park H Park S Lim JS Lim SY Transmission and infectious SARS-CoV-2 shedding kinetics in vaccinated and unvaccinated individuals. JAMA Netw Open. 2022;5(5):e2213606. 10.1001/jamanetworkopen.2022.13606 35608859 PMC9131744

[52] Knobloch JK Franke G Knobloch MJ Knobling B Kampf G . Overview of tight fit and infection prevention benefits of respirators (filtering face pieces). J Hosp Infect. 2023;134:89-96. 10.1016/j.jhin.2023.01.009 36738992 PMC9894678

[53] Jones RM Bleasdale SC Maita D Brosseau LM CDC Prevention Epicenters Program . A systematic risk-based strategy to select personal protective equipment for infectious diseases. Am J Infect Control. 2020;48(1):46-51. 10.1016/j.ajic.2019.06.023 31358421 PMC7132808

[54] Chughtai AA Chen X Macintyre CR . Risk of self-contamination during doffing of personal protective equipment. Am J Infect Control. 2018;46(12):1329-34. 10.1016/j.ajic.2018.06.003 30029796

